# Supplementary material for: Performance replication of the Hospital Mental Health Risk Screen in ethnoracially diverse U.S. patients admitted through emergency care
Source: PLoS One. 2024 Oct 1;19(10):e0311256. doi: 10.1371/journal.pone.0311256 (PMC11444411; doi:10.1371/journal.pone.0311256)
Supplement: S1 File — (PDF) [file pone.0311256.s001.pdf]

Hospital Mental Health Risk Screen

Answers to these questions show how likely mental health problems are in the next few months.
Check a box for each question to say how often each happened to you.

	Never	Less than once a year	A few times a year	A few times a month	At least once a week	Almost every day or more
In your day-to-day life, how often have you been treated with less respect than other people?	☐ 0	☐ 1	☐ 2	☐ 3	☐ 4	☐ 5

	None of the time	A little of the time	Some of the time	About half of the time	More than half the time
In the past, how often has feeling anxious, nervous, down or depressed kept you from enjoying life?	☐ 0	☐ 1	☐ 2	☐ 3	☐ 4
Since you came to the hospital, how often has each of these happened to you?					
Feeling cut off or isolated from other people.	☐ 0	☐ 1	☐ 2	☐ 3	☐ 4
Feeling very stressed.	☐ 0	☐ 1	☐ 2	☐ 3	☐ 4
Things around you seemed strange or unreal.	☐ 0	☐ 1	☐ 2	☐ 3	☐ 4
Feeling bad about yourself – or that you are a failure or have let yourself or your family down.	☐ 0	☐ 1	☐ 2	☐ 3	☐ 4
In situations when you used to have good feelings like happiness or love, you didn't.	☐ 0	☐ 1	☐ 2	☐ 3	☐ 4
Feeling really pessimistic about yourself, other people, or the way the world is.	☐ 0	☐ 1	☐ 2	☐ 3	☐ 4
Being very aware and nervous about what was going on around you.	☐ 0	☐ 1	☐ 2	☐ 3	☐ 4
In the next month, how much do you…					
… expect to feel that you aren't on top of things?	☐ 0	☐ 1	☐ 2	☐ 3	☐ 4

****

Hospital Staff: Add the numbers below checked boxes to obtain the total score: _________
