# Supplementary material for: Performance replication of the Hospital Mental Health Risk Screen in ethnoracially diverse U.S. patients admitted through emergency care
Source: PLoS One. 2024 Oct 1;19(10):e0311256. doi: 10.1371/journal.pone.0311256 (PMC11444411; doi:10.1371/journal.pone.0311256)
Supplement: S2 File — (PDF) [file pone.0311256.s002.pdf]

Examen Hospitalario de Riesgos Para La Salud Mental

Las respuestas a estas preguntas muestran la probabilidad de que tenga problemas de salud mental en los próximos meses. Marque una casilla junto a cada pregunta para indicar con qué frecuencia le sucedieron estas situaciones.

	Nunca	Menos de un vez al año	Varias veces al año	Varias veces al mes	Al menos una vez a la semana	Casi todos los días o más
En su vida cotidiana, ¿con qué frecuencia lo han tratado con menos respeto que a otras personas?	☐ 0	☐ 1	☐ 2	☐ 3	☐ 4	☐ 5

	Nunca	Un poco del tiempo	Algunas veces	Cerca de la mitad de las veces	Más de la mitad del tiempo
En el pasado, ¿con qué frecuencia el hecho de sentirse ansioso, nervioso, decaído o deprimido le ha impedido disfrutar de la vida?	☐ 0	☐ 1	☐ 2	☐ 3	☐ 4
Desde que llegó al hospital, ¿con qué frecuencia le ha ocurrido cada una de estas cosas?					
Sentirse apartado o aislado de los demás.	☐ 0	☐ 1	☐ 2	☐ 3	☐ 4
Sentirse muy estresado.	☐ 0	☐ 1	☐ 2	☐ 3	☐ 4
Las cosas a su alrededor parecen extrañas o irreales.	☐ 0	☐ 1	☐ 2	☐ 3	☐ 4
Sentirse mal con si mismo, o pensar que es un fracaso o que ha defraudado a sí mismo o a su familia.	☐ 0	☐ 1	☐ 2	☐ 3	☐ 4
En situaciones que solía tener buenos sentimienos, como felicidad o amor, ya no los siente .	☐ 0	☐ 1	☐ 2	☐ 3	☐ 4
Sentirse muy pesimista sobre si mismo, sobre los demás o sobre cómo es el mundo.	☐ 0	☐ 1	☐ 2	☐ 3	☐ 4
Estar muy atento y nervioso por lo que ocurre a su alrededor.	☐ 0	☐ 1	☐ 2	☐ 3	☐ 4
En el próximo mes, ¿cuánto espera...					
...sentir que no está al tanto de las cosas?	☐ 0	☐ 1	☐ 2	☐ 3	☐ 4

****

Personal hospitalario: Sume los números de las casillas marcadas para obtener la puntuación total: _________
