# Supplementary material for: Performance replication of the Hospital Mental Health Risk Screen in ethnoracially diverse U.S. patients admitted through emergency care
Source: PLoS One. 2024 Oct 1;19(10):e0311256. doi: 10.1371/journal.pone.0311256 (PMC11444411; doi:10.1371/journal.pone.0311256)
Supplement: Attachment — Submitted filename: Response to PLOS One reviews of HMHRS Replication MS 8-6-24.docx [file pone.0311256.s003.docx]

**Response to Reviewer Comments**

Thank you for the opportunity to revise and resubmit this manuscript. We have addressed all of the reviewer concerns below and in the manuscript. We have uploaded the revised manuscript with and without tracked changes. We did not track formatting changes made to bring the manuscript into compliance with PLOS One style (e.g., larger font size for headers, removing degrees for authors). Page numbers below refer to pages in the manuscript that does not include tracking.

**Reviewer #1:**

1) More information is needed regarding how participants were approached/recruited, including the proportion approached who agreed to participate.

On lines 149-157, we have added details on how participants were approached/recruited, information provided to them, and payments. On lines 212-213, we reported on the timing of the screen administration (mean days) relative to admission. Unfortunately, we were not able to track patients approached who declined to participate, so we cannot provide that information. We have added this as a limitation to the study on lines 342-343.

2) Table 2 should present confidence limits for sensitivity, specificity, PPV, and NPV.

Thank you for this suggestion. We have added the confidence intervals for SE, SP, PPV, and NPV to the table and agree that this provides valuable information about the results. We also moved the SE and SP information for injury patients and for acute illness patients into Table 2 and added AUC, PPV, NPV and confidence intervals to provide these details for those two groups.

3) Using the total score of PHQ8, GAD7, and SPTSS as an indicator of mental health need seems (at least to me unusual). Is there some evidence or rationale supporting this approach? How was the cut-point of 37 selected (and was it selected in advance)? This sum score would seem to give greater weight to the SPTSS than to the PHQ8 and GAD7 combined.

We appreciate that using a dimensional measure is somewhat novel, and we have added an explanation on lines 107-115 in the introduction to explain the rationale for this choice. In short, we chose this approach because of the advantages of dimensional measures over diagnostic taxonomies and because responses to trauma cross diagnostic boundaries and are highly comorbid. Specifically, PTSD, depression, and anxiety disorders are highly comorbid with past research showing about 50% of those with PTSD also meeting diagnostic criteria for depression and 20% also meeting diagnostic criteria for anxiety disorder. Since the goal of screening is to identify patients with mental health risk, a focus on diagnosis seems like it would not serve patients who have symptoms from different diagnostic categories that collectively warrant clinical attention. Similarly, when the cumulative impact of symptoms is the focus, having more items that are considered symptoms of PTSD does not seem problematic.

4) The discussion should address whether a new screening tool is actually needed (rather than simply relying on well-established tools such as the PHQ2, GAD2, and the PC-PTSD-5.

On lines 78-84, we have explained that we developed a measure of mental health risk because the presence of symptoms in the days following exposure to traumatic stress do not accurately identify which patients will develop mental health problems. To further clarify, on lines 81-85, we have provided an example of screening for presence vs risk of breast cancer that is familiar in medical settings.

**Reviewer #2:** The current study aimed to replicate the predictive performance of the Hospital Mental Health Risk Screen (HMHRS) in a new sample of patients from three hospitals in the United States. Replication studies are crucial for validating the effectiveness and reliability of assessment tools across different patient populations. This study is straightforward and generally well-described. Methods are appropriate. I have the following comments for the authors.

1. HMHRS was created as a screening tool to identify mental health risks in patients following a traumatic event. What was the reason for having a follow-up period of two months after admission?

We have added an explanation on lines 134-138 in the methods section of the reason for assessing symptoms at two months post-admission. Our prospective research on recovery after traumatic injury showed that, for the vast majority of those who recovered well, initial elevations in symptoms decreased to normal levels by 6 weeks. In addition, our pilot work that was the basis for the development of the HMHRS predicted posttraumatic symptoms at the 2-month time point.

2. “Using this definition, an estimated 99.9% of the population of patients who did not have elevated levels of depression, anxiety, or PTSD symptoms at follow-up would be categorized as “not high” on Mental Health Symptoms.” How is the 99.9% estimation done?

We expanded the explanation for how we identified the cut score for Mental Health Symptoms on lines 185-193. We identified the patients who did not report elevated depression, PTSD, or anxiety symptoms at follow-up by applying cut scores for the PHQ-8 and GAD-7 used in primary care and the cut score for the SPTSS based on prior research. The mean score for Mental Health Symptoms for patients with no symptom elevations was 10.5 (SD = 8.8). In a normal distribution, 99.7% of data points fall within one SD of the mean, and 99.85% of data points fall at or below the value of 3 SDs above the mean. Therefore, a score of 37, which falls 3 SDs above the mean of 10.5, was estimated to represent 99.9% of the population of patients who did not have elevations in symptoms.

3. The manifestation of PTSD can exhibit significant variation among individuals, including immediate onset, acute onset, and delayed onset. Performing HMHRS screening immediately after emergency care may be inadequate in identifying individuals who have developed acute or delayed onset PTSD.

A 2007 systematic review of research indicated that delayed onset in the absence of prior symptoms was rare, and these cases could better be understood as exacerbations or reactivation of prior symptoms. See Andrews et al. 2007: <https://web.archive.org/web/20190716195953id_/https://ajp.psychiatryonline.org/doi/pdf/10.1176/appi.ajp.2007.06091491>

These findings were also reflected in a change to the diagnostic criteria for PTSD from allowing a specifier of “Delayed Onset” in DSM-IV to “With delayed expression” in DSM-5. The new specifier applies when “full diagnostic criteria are not met until at least 6 months after the event (although the onset and expression of some symptoms may be immediate).”

In addition to these changes, we also updated the comparison of performance with other screens on lines 295-302 to refer to a 2021 publication (vs a 2018 publication) and noted that training is required to administer and score the ITSS. And we added an author, Mallory Williams, who worked on the study and contributed to the manuscript, but had moved and we were unable to reach him at the time of the prior submission. We since were able to get his input on the original and the revised manuscript.
