# Supplementary material for: Performance replication of the Hospital Mental Health Risk Screen in ethnoracially diverse U.S. patients admitted through emergency care
Source: PLoS One. 2024 Oct 1;19(10):e0311256. doi: 10.1371/journal.pone.0311256 (PMC11444411; doi:10.1371/journal.pone.0311256)
Supplement: Attachment — Submitted filename: Response to reviews HMHRS Replication MS 8-27-24.docx [file pone.0311256.s004.docx]

**Response to Reviewer Comments**

Thank you for the opportunity to revise and resubmit this manuscript. We have addressed all of the reviewer concerns below and in the manuscript. Page numbers below refer to pages in the manuscript that does not include tracking.

**Reviewer comments and responses:**

1. The description regarding how participants were identified and recruited is not adequate. Specifically: How were potential patients identified (from admission logs, health records, etc.). What initial eligibility criteria were applied? Was every eligible patient approached - or how were patients selected to be approached? The detail here is absolutely essential, and these findings have no value or meaning without that detail.

Apologies for not including this detail in the prior revision. We agree that these details are important for readers to know so they can interpret the study results. On lines 153-158, we added that eligible patients were identified through electronic medical records, that patients were included who were treated in the emergency department and admitted for inpatient care, and that research staff attempted to approach all eligible patients who were able to answer study questions. An additional detail that we could include, but that seems unnecessary is that some patients were discharged before a staff member could recruit them.

2. The discussion does not clearly address why a new screening questionnaire is needed. It's notable that the criterion or "gold standard" in this study was a combination of screening questionnaires already in widespread use. Why would this new questionnaire be superior to or preferred over the PHQ-2/9, GAD-2/7, and the SPTSS (or PCL-5). Given the extensive data documenting the acceptability, accuracy, and utility of those measures (including data regarding performance across a wide range of people and settings), it is necessary to identify any clear advantage of this new measure.

This issue was covered in more detail in our prior paper on the development of the measure, but we understand why it is important to address in the replication paper as well. We have added an explanation on lines 78-88 to clarify that measures of PTSD, depression, and anxiety have been developed and validated to assess mental health conditions at the time of the assessment, but were not designed to predict future mental health symptoms and have not shown promise of performing well to identify mental health risk. We cite specific examples of mixed and poor performance of the PCL, the PC-PTSD, and the PHQ-8 to predict later PTSD symptoms. These findings are consistent with a larger literature on Acute Stress Disorder that shows symptoms in the days after an event do not accurately predict later mental health problems. Also, Jensen et al. (2022) (citation #21 in the paper) reviewed a large number of studies of screening tools (including the PCL, PC-PTSD, and many others) and found that no brief, self-report screen screens have reliably performed well in the U.S.. In addition, no screens have been developed for or studied in acute illness patients or in U.S. ethnoracial subgroups.
