# Supplementary material for: Performance replication of the Hospital Mental Health Risk Screen in ethnoracially diverse U.S. patients admitted through emergency care
Source: PLoS One. 2024 Oct 1;19(10):e0311256. doi: 10.1371/journal.pone.0311256 (PMC11444411; doi:10.1371/journal.pone.0311256)
Supplement: Attachment — Submitted filename: Response to reviews HMHRS Replication MS 9-12-24.docx [file pone.0311256.s005.docx]

**Response to Reviewer Comments**

Thank you for the opportunity to revise and resubmit this manuscript. We have addressed the remaining reviewer concern below and in the manuscript.

**Reviewer comments and responses:**

Reviewer #1: The claim that existing screening tools "have not shown promise of performing as brief screens for mental health risk" should either be supported by evidence or deleted.

Apologies that it was not clearer in the manuscript that the evidence to support this statement is provided in the two sentences that follow it (lines 82-88). To further clarify that the sentences that follow provide evidence to support the statement on line 81-82, we have simplified the statement and included a transition phrase “Specifically” at the start of the next sentence. We also added words in the description of supporting evidence (lines 82-88) to characterize the levels of sensitivity and specificity as poor, low, or good for readers who may not be familiar with this index of accuracy in prediction.
